# Epidemiological Study of Pesticide Poisoning in Domestic Animals and Wildlife in Portugal: 2014–2020

**DOI:** 10.3389/fvets.2020.616293

**Published:** 2021-01-14

**Authors:** Andreia Grilo, Anabela Moreira, Belmira Carrapiço, Adriana Belas, Berta São Braz

**Affiliations:** CIISA-Centro de Investigação Interdisciplinar em Sanidade Animal, Faculdade de Medicina Veterinária, Universidade de Lisboa, Lisboa, Portugal

**Keywords:** pesticides, poisoning, wild and domestic animals, Portugal, animal welfare

## Abstract

Nowadays the intentional poisoning of domestic and wild animals is a crime in the European Union (EU), but as in the past the poison is still used in rural areas of a number of European countries to kill animals that were considered harmful for human activities. From January 2014 up until October 2020, the Laboratory of Pharmacology and Toxicology of the Faculty of Veterinary Medicine (LFT-FMV) has done the analytical detection of poisoning substances in 503 samples of wildlife and domestic animals and pesticides residues were found in 239 of the samples analyzed. In this retrospective study, toxicology results from domestic species (dog, cat, sheep, cows, and horses), wildlife species (red foxes, birds of prey, lynx, and wild boar), and food baits, are presented. During this period the samples analyzed at the LFT-FMV, were received from all over the country. Analytical detections were performed via solvent extraction followed by thin layer chromatography. Molluscicides (47%, *n* = 109) and Carbamates (24%, *n* = 57) were found to be the first category of pesticides involved in intoxications, in both domestic and wild animals, followed by rodenticides (13%, *n* = 30)—in this group second and third generation, were the most represented; Strychnine is the third (11%, *n* = 26) even though this pesticide has been banned in Portugal since 1988 and in the European Union since 2006 and finally Organophosphates (5%, *n* = 11) in the small number. This study allowed to realize that a great number of positive samples involved banned pesticides (i.e., Aldicarb and Strychnine) but, at the same time, many positives cases were due to the exposure to commercially available products (i.e., Methiocarb and Anticoagulant rodenticides). Also, it's possible to identify the areas where domestic species are the most affected (i.e., Setubal and Lisboa) and the areas where the wild animals are the mainly affected species (i.e., Faro, Castelo Branco, and Bragança).

## Introduction

Poisoning of wild and domestic animals is a problem in all the countries in the world that has been described by several authors from ancient times to nowadays (1–16).

Acute pesticide poisonings of wildlife and domestic animals can occur due to accidents with substances approved for several uses, and due to deliberate and illegal attempt to poison animals even with baits, and by secondary poisonings (17).

Pesticides or more correctly Biocides, group include a wide range of chemicals, with a great diversity of applications, including agriculture and domestic applications. However, pesticides are often implicated in acute intoxications, in particular insecticides like molluscicides (6, 7, 17, 18).

The occurrence of poisonings related to pesticides is influenced by many factors, such as the availability of the substances without restrictions to the acquisition, the possibility to buy them easily, the agricultural techniques, the cultural background and the knowledge and instruction of users, etc (9). In general, and in most of the countries, pesticides are easy to purchase not only for professional uses but also for domestic applications since in some of these countries it's possible to get them at supermarket and at small shops dedicated to agriculture products and as said before on the online market.

One of the best examples is strychnine and aldicarb. These substances had been banned from the market for use in the agriculture and as a rodenticide (strychnine), in Portugal in 1988 and in the European Union in 2006 for strychnine (19), and in 2003 for aldicarb (20) but both are still frequently found as responsible by intentional and accidental intoxications (21, 22). Considering these facts the continuous collection of epidemiological data on animal poisoning in order to obtain useful information on toxicant trends to carry out and enhance preventive measures for an appropriate risk management, to help veterinarians to manage cases of suspected poisoning, and finally to draw attention toward an issue that has also environmental and human health implications is of crucial importance (4).

In this study, the incidence of pesticides detection from animals/carcasses, baits, and other materials sent to LFT-FMV for toxicological evaluations was analyzed, trying to provide a general overview on animal exposure to pesticides, in both domestic and wild species, for a period of the last 6 years.

## Materials and Methods

### Area of Study and Collection of Samples

Portugal has an enormous geophysical diversity, which provide an important habitat for wildlife mostly on border with Spain on the North, Centre and South of the country, where lot of birds of prey can be found.

In all the country but specially in the North and South of the country there several areas where animal protection programs, such as Life Rupis, are running.

Since 2003 Portugal have a multidisciplinary program (PROGRAMA ANTIDOTO) to study and understand which and how toxic substances are used, in order to stop animals killing, particularly wild animals (Griffon vultures, red and black kites, foxes, wolves), but also domestic species that can be exposed to these substances and be intoxicated. This program recently has a great improvement by the creation of a network of necropsy centers and laboratories of Toxicology (LFT-FMV is one of these laboratories) that in conjugation with all the other entities involved try reduced animal poisoning in Portugal. So, in case of the identification of a poison bait and suspected poisoned animal, the competent/policies authority (SEPNA/GNR or PSP) must be informed and those materials should be sent to a laboratory service for necropsy and/or toxicological investigations in order to identify the potentially toxic substance present. If the poisoning events are confirmed, the competent authority has to be notified and legal prosecution may follow.

The analyzed samples include biologics matrixes of the different species (blood, liver, gastric, and intestinal contents), and baits. These samples come from ANTIDOTO Program, but also from private requests for example from veterinary practitioners.

Data analysis included the location of the poisoning, type of species and number of animals affected, and the pesticide(s) detected.

### Analytical Methodology

Procedure for the extraction, purification, and detection of pesticides in samples from animal poisoning incidents have been developed and validated in LFT-FMV. The extraction was performed using specific solvents for each group, followed by separation and characterization by thin layer chromatography (TLC) in Merck silica gel G 60 F254.

The detection of all substances was carried out by qualitative TLC, all the plates were spotted with the extract of the sample and the corresponding standards which allows to have always a positive control in each analysis. In addition to this, the measurement of the retention factor (Rf) of each compound was done and compared with the Rf of the sample (23, 24).

*For Strychnine* the sample was minced, mixed in aqueous solution (water and amonia, pH 9/10), and agitated with a magnetic stirrer during at least 3 h. After, the sample is decanted and a purification step is performed to remove coextractants with chloroform, after that the final step of the extraction procedure is a liquid-liquid extraction. The phase with chloroform was collected to a round bottom flask and evaporated until a dry extract was get. The final concentrated extract was spotted on a silica gel TLC with a mobile phase chloroform/methanol (9.5:0.5) (25). After plate developing, the detection of strychnine was performed by spot visualization at 254 nm and after spraying an acidic solution of iron chloride and perchloric acid, which produce a red complex with strychnine when the plates are heated to 110 degrees about 10 min (26).

*For Coumarinics* the sample was also minced, but mixed in a solution of acetone and chloroform, and agitated with a magnetic stirrer during at least 3 h. After this period, the sample is decanted, and liquid-liquid extraction was made with water addition. Then the aqueous phase was discharged, and the organic phase was collected to a round bottom flask and evaporated until a dry extract was get. The final concentrated extract was spotted on a silica gel TLC with a mobile phase n-hexane/acetone (9:1) (25). After plate developing, the detection of coumarinics (Bromadiolone. Brodifacoum, Difenacoum and Coumatetralyl) was performed by spot visualization at 254 nm, spraying an 2% Sodium hydroxide and etanol solution, which produced a fluorescence when viewing under UV light (wave-length 366 nm) (27).

*Finally, for Organophosphates/Carbamates and molluscicides*, the minced sample was mixed with acetonitrile, and agitated with a magnetic stirrer during at least 3 h. After this period, the sample is decanted, and liquid-liquid extraction was made with n-hexane addition. Then the n-hexane phase was collected to a round bottom flask and evaporated until a dry extract was get. The final concentrated extract was spotted on a silica gel TLC with a mobile phase n-hexane/acetone (9:1) (25). After plate developing, the detection of organophosphates was performed by spraying a palladium dichloride and hydrochloric acid solution, which produce a yellow/Brown complex with the substances of this group when the plates are heated to 110 degrees about 10 min. The carbamates detection was performed by spraying a zinc chloride and diphenylamine in acetone solution, which produce a bluish-green complex in the presence of carbamates when heated to 110 degrees about 10 min. The molluscicides detection was performed by spraying an p-anisaldehyde solution, which produced a pink complex for methiocarb and a purple complex for methaldeyde when heated to 110 degrees about 5 min. To all the substances a visualization under UV light at 254 nm was performed to mark the spots before the spraying (23).

For all the groups, including anticoagulants (brodifacum, bromadiolone, coumatetralyl), the detection limit is 2 μg.

The analysis of pesticides in suspected samples was carried out, if possible, according to symptomatic and pathological evidence, data provided by veterinarians in the reports sent with the animals for necropsy or with the baits, in addition to epidemiological data (poisons normally used in the geographical area in question for example).

The great difficulty faced in these cases is to distinguish whether they are intentional or accidental poisonings.

### Statistical Analysis

Data collected were organized in a database of Microsoft Office Excel® and descriptive analysis and graphics were developed and created also with Microsoft Office Excel®. Descriptive data are reported in absolute and percentual values (24).

## Results

### Positive Samples

From the total number of 503 samples received between January 2014 and October 2020 with intoxication suspicion by pesticides the requests come from ANTIDOTO program (animal protectionist groups, public health authorities, regional and central bodies as local police departments) and from private entities (personal requests, veterinary practitioners).

**Figure 1:**
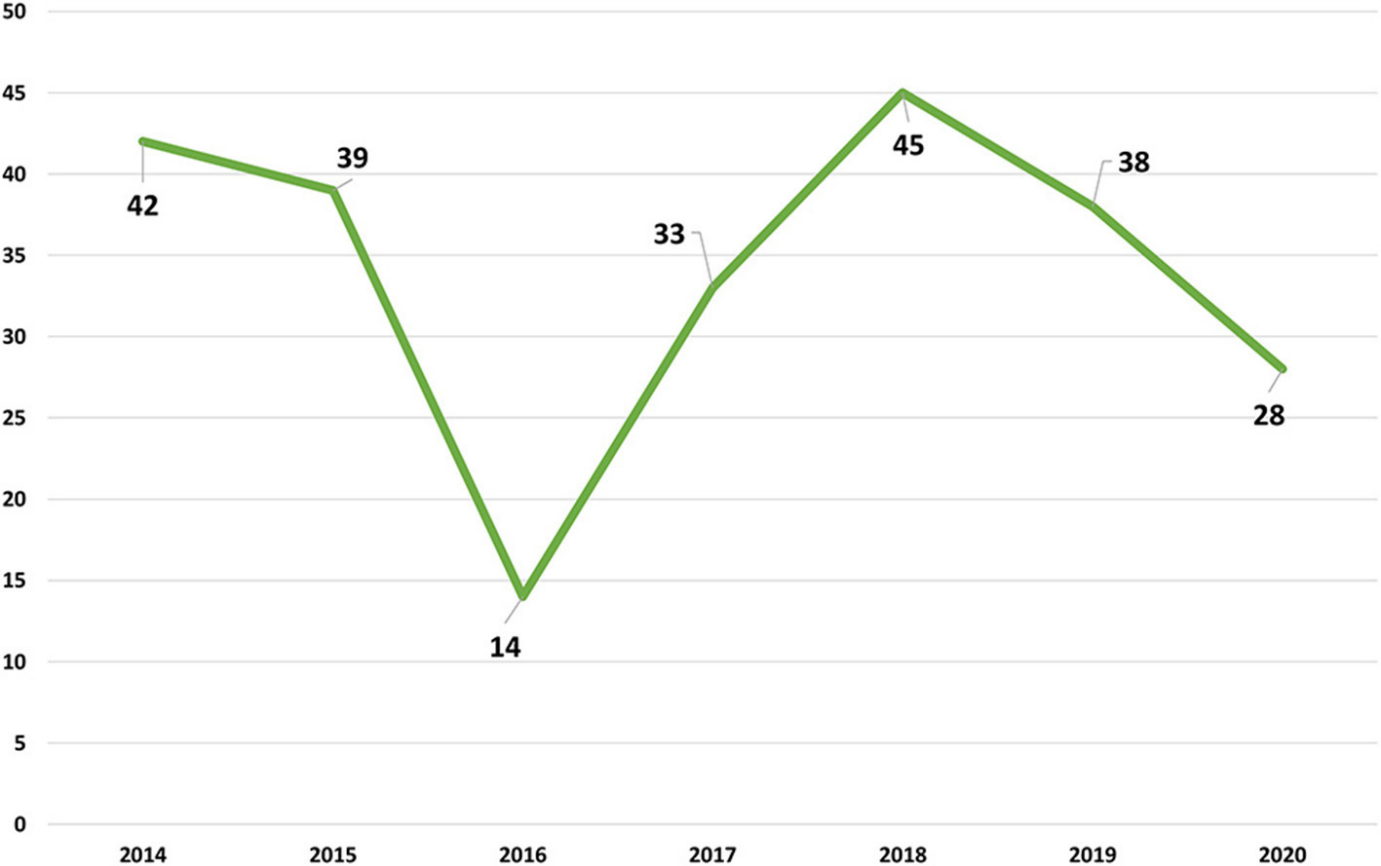
Evolution of positive samples per year between 2014 and 2020.

In the Figure 1 the evolution of the number of positive cases between 2014 and 2020. The number of positive samples is very similar in the several years with the exception of 2016 with a lower number that reflects the lower availability of LFT-FMV to receive samples and to do the respective analysis.

**Figure 2:**
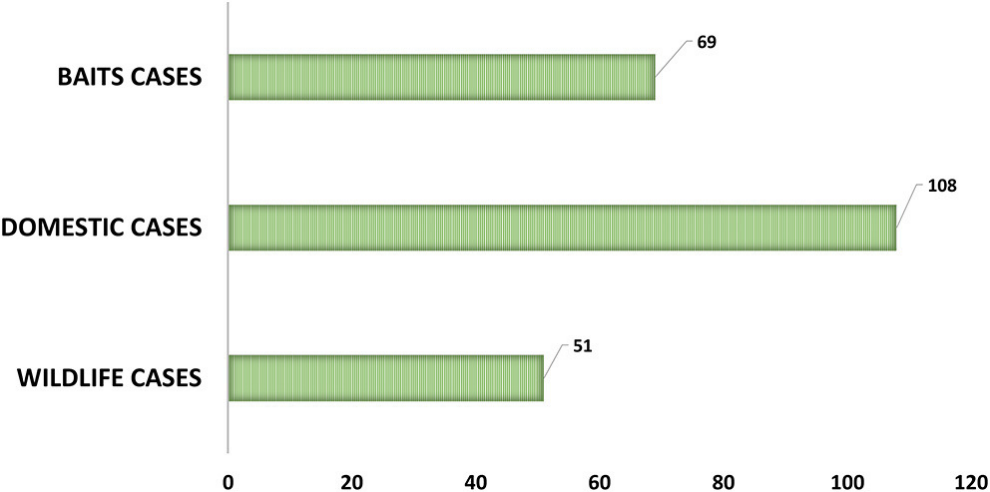
Number of positive cases by domestic, wildlife, and baits samples.

**Figure 3:**
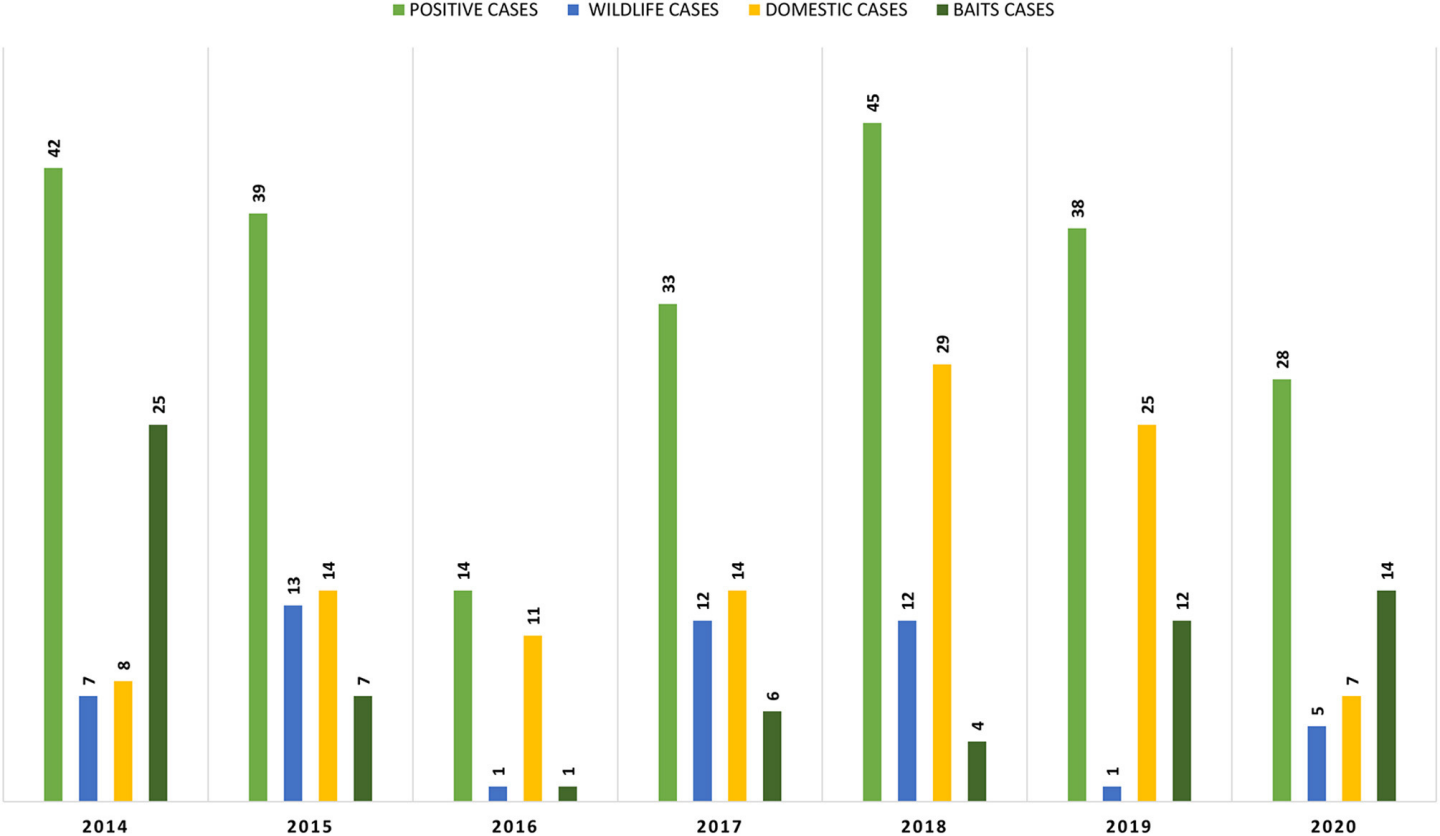
Positive cases in domestic, wildlife, and baits samples by year.

As can be observed in Figure 2, from the 239 positive cases, 108 are from domestic species, 69 from baits and 51 from wild species. The evolution of these cases, by type and by year can be observed in Figure 3.

Concerning baits, most of the samples received over the years came alone and not associated with a specific animal species. But from the 69 positive cases of baits, 15 are associated with foxes, 6 with dogs and finally 2 with cats, for all the others no relationship could be established.

### Geographical Areas of Samples

**Figure 4:**
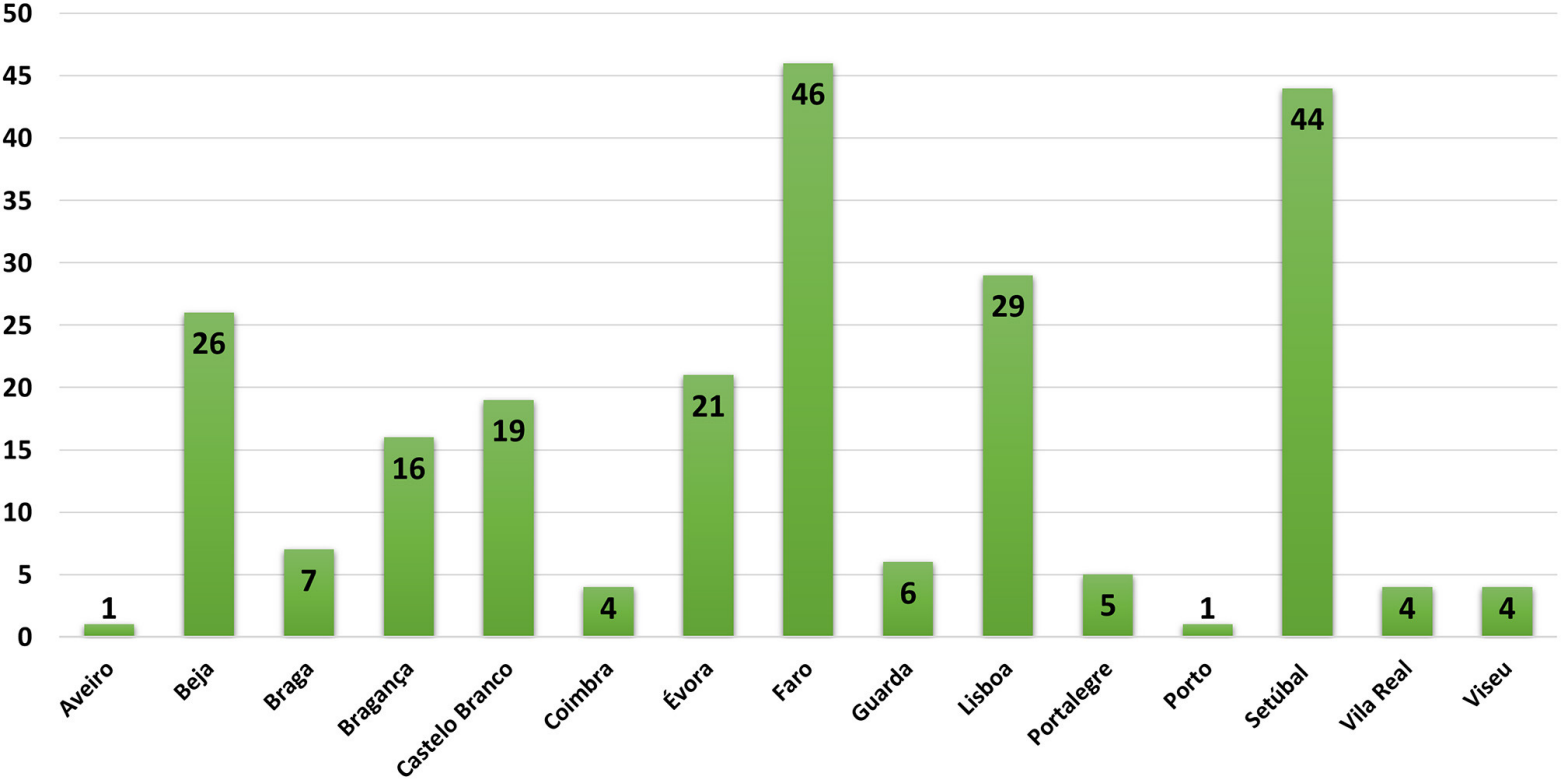
Geographic distribution of cases.

Portugal is divided into three main regions, North, Center, and South that includes 18 districts. Data obtained on this study allows the distribution of the results in two major groups. In the first one samples for pesticide detection comes from the districts of Setubal and Lisbon and includes the group where the most affected species are the domestic animals (30.5%, *n* = 73). In the other group wild animals are those mainly affected, and samples comes from the districts of Faro, Castelo Branco and Bragança (34%, *n* = 81). These results are showed in the Figure 4.

### Detected Pesticides

**Figure 5:**
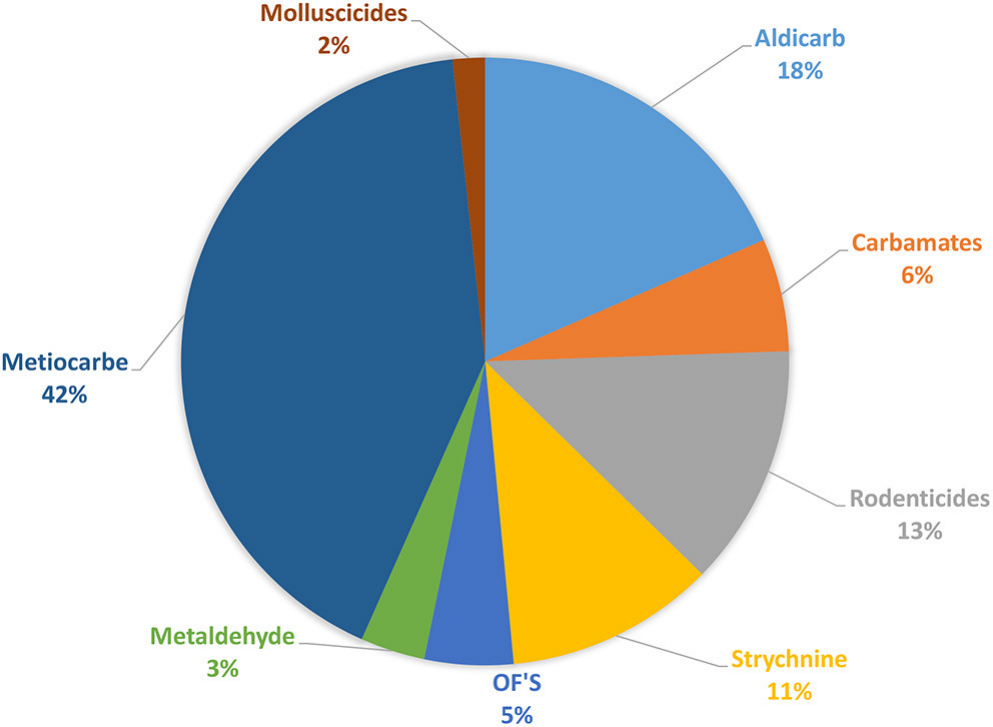
Distribution of detected substances by group of pesticide.

Molluscicides (47%, *n* = 109) and Carbamates (24%, *n* = 57) were found to be the first category of pesticides detected in samples of suspicious intoxications, in both domestic and in wild animals, followed by rodenticides, with first and second generation represented (13%, *n* = 30), by Strychnine (11%, *n* = 26) and in the end by Organophosphates (5%, *n* = 11). This total distribution by group of pesticide can be observed in the Figure 5. The distribution of the substances detected, by domestic animals, by baits and by wild species can be observed in **Figures 8–10**, respectively.

### Affected Species

Results obtained also allow the identification of the most affected animals, either in wild and in domestic species.

**Figure 6:**
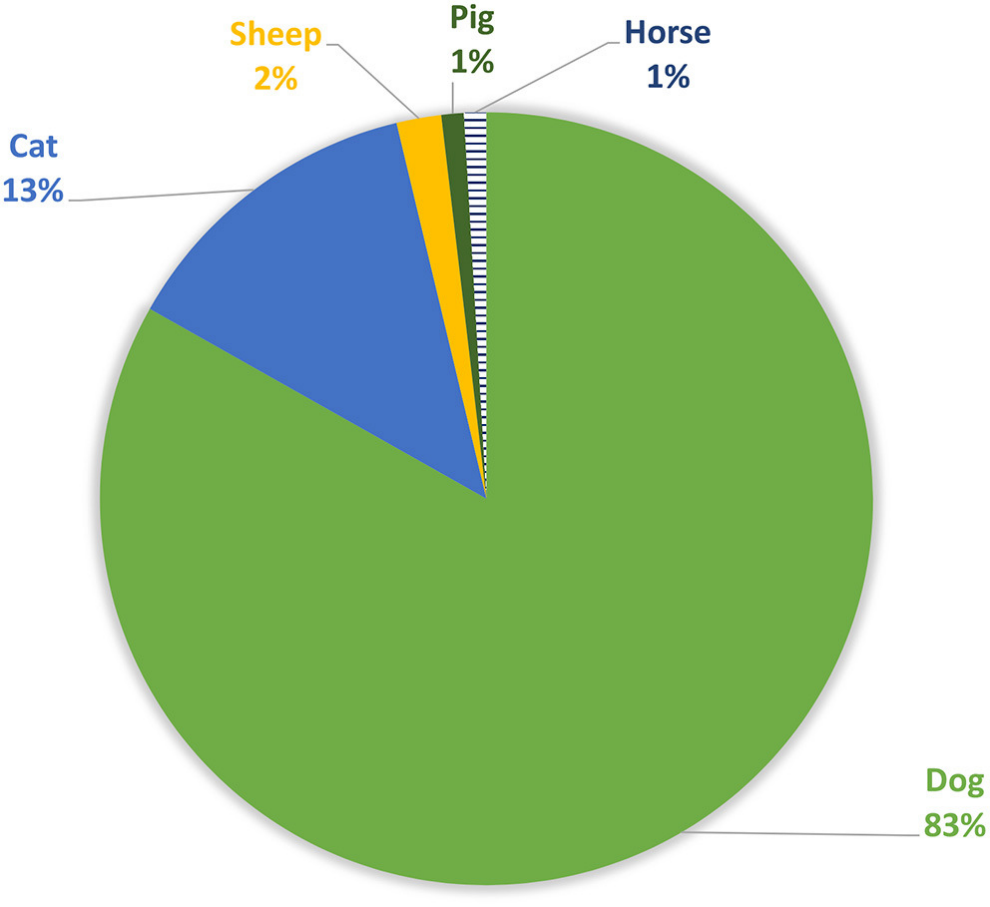
Domestic species affected.

The most affected domestic animal species (Figure 6) are mainly dogs (83%, *n* = 89), cats in lower number (13%, *n* = 14) and a small amount of other species (sheep, horses and pigs) (4%, *n* = 4).

**Figure 7:**
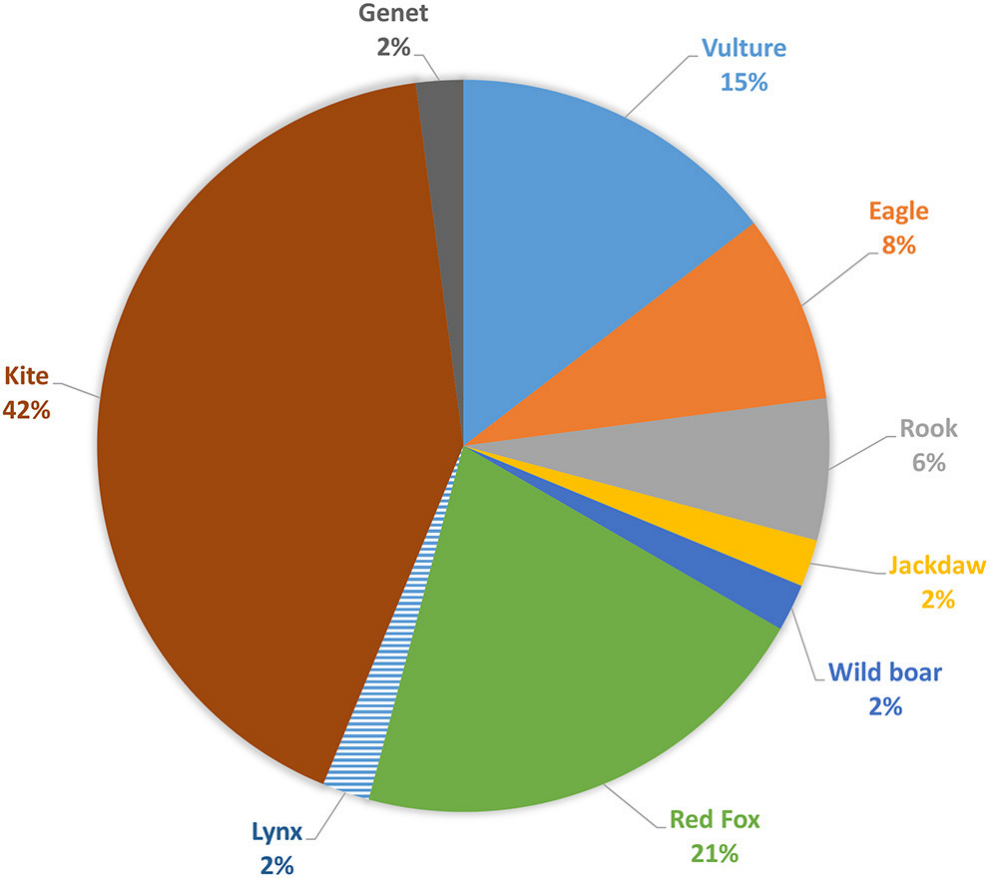
Wild species affected.

Concerning wild species (Figure 7), the most affected animals are kites (42%, *n* = 20), red foxes (21%, *n* = 10), vultures (15%, *n* = 7), eagles (8%, *n* = 4), rooks (6%, *n* = 3), and a small amount of other species (6%, *n* = 4).

## Discussion

Results obtained in this study show that Molluscicides and Carbamates intoxication are still one of the major causes of poisoning in domestic and wild species in Portugal following the pattern previously reported by our team (8, 28).

Portugal has favorable climatic conditions for snails and slugs. Baits are used in domestic and commercial applications to protect gardens and crops. Although a variety of methods and chemicals have been used to control these pests, the most commonly used preparations are bran-based powders, flakes, granules, or pellets containing metaldehyde (metacetaldehyde) or methiocarb (3,5-dimethyl4-methyl-mercaptophenyl-N-methyl-carbamate) for distribution on the ground (3, 5, 7, 10, 11). Molluscicides can poison domestic animals and wildlife, including birds, which are natural mollusc predators and also can eat baits made with these substances. What makes this group the most used in intentional poisonings, is the fact that these compounds are available on commercial surfaces (supermarkets, DIY stores) without great control over purpose or quantities purchased, greatly facilitates these crimes being succefull.

Insecticides are used worldwide, and it is inevitable that accidental poisoning of animals occur. Organophosphate and carbamates are highly toxic to all animals; some organophosphates can induce violent convulsions and kill in few minutes. These chemicals cause poisoning in animals by inhibiting the enzyme, acetylcholinesterase (AChE) which normally functions to degrade acetylcholine in nerve synapses.

Recently in in Portugal the carbamate Aldicarb has been detected again compared to previous years (23). Aldicarb, was introduced in agriculture in the 1960s with the aim of controlling a wide variety of insects, mites and nematodes. The substance is presented in the form of small black granules. When is possible to find the bait associated with this substance it is easily detectable with the naked eye, but no smell or taste can be perceived by the animal and the intoxication is facilitated (29). The recent use of this substance verified again because pesticide was detected in baits and wild animals (**Figures 9, 10**), can occur due to amounts of obsolete product that may be present in private farms which have not been destroyed and could be motivating farmer's intentional use against domestic and wild fauna.

Strychnine, an indole alkaloid obtained from the seeds of the tree *Strychnos nux-vomica* or *Strychnos ignatii*, is mostly used to poison rats, birds, and wild carnivores such, foxes, and wolves. Strychnine is an extremely toxic, fast-acting poison which acts on the central nervous system leading to convulsions and ultimately death via respiratory failure. The recovery of strychnine alkaloid from the stomach contents, blood, vomitus, liver, urine, and kidneys confirms the diagnosis. Although stomach contents or vomitus have the highest concentration if death was rapid, but residues are present in liver tissues over a long time, what make this organ the ideal tissue for its detection.

Strychnine usage and commercialization has been banned since 1988; however, strychnine continues to be one of the used substances for intentional poisoning in Portugal as results (in Figure 5) shows, small amounts of strychnine main still remain in storage, or street drugs with strychnine may be acquired. The apparent lack of control over the possession of this pesticide means that many people still have it and use it (3, 5, 7, 10, 11). As prohibited or unauthorized use have been detected, even though they are not for sale, this evidence also suggests that there may be illegal sales of these substances online (30).

**Figure 8:**
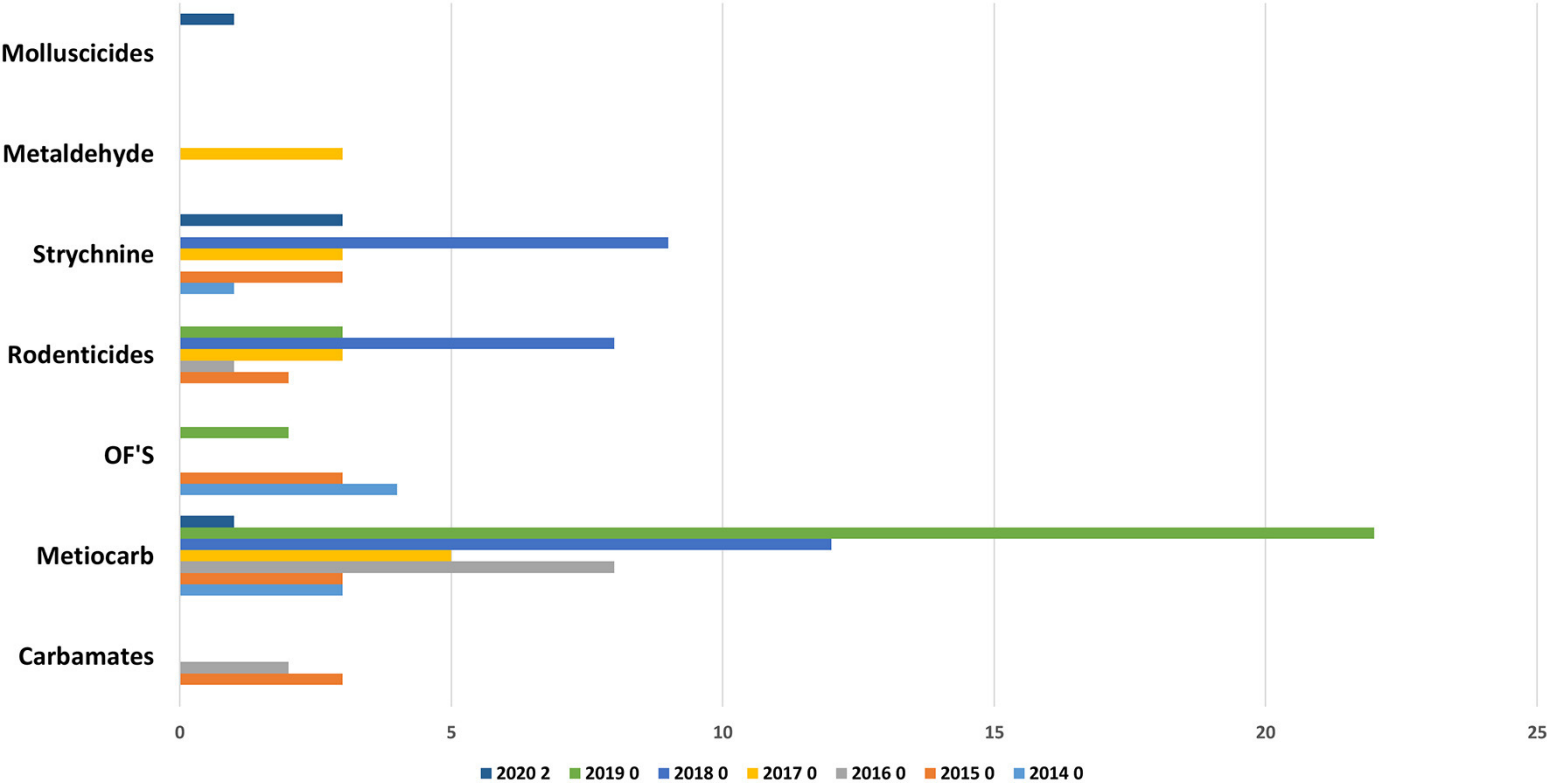
Distribution of substances in domestics per year.

**Figure 9:**
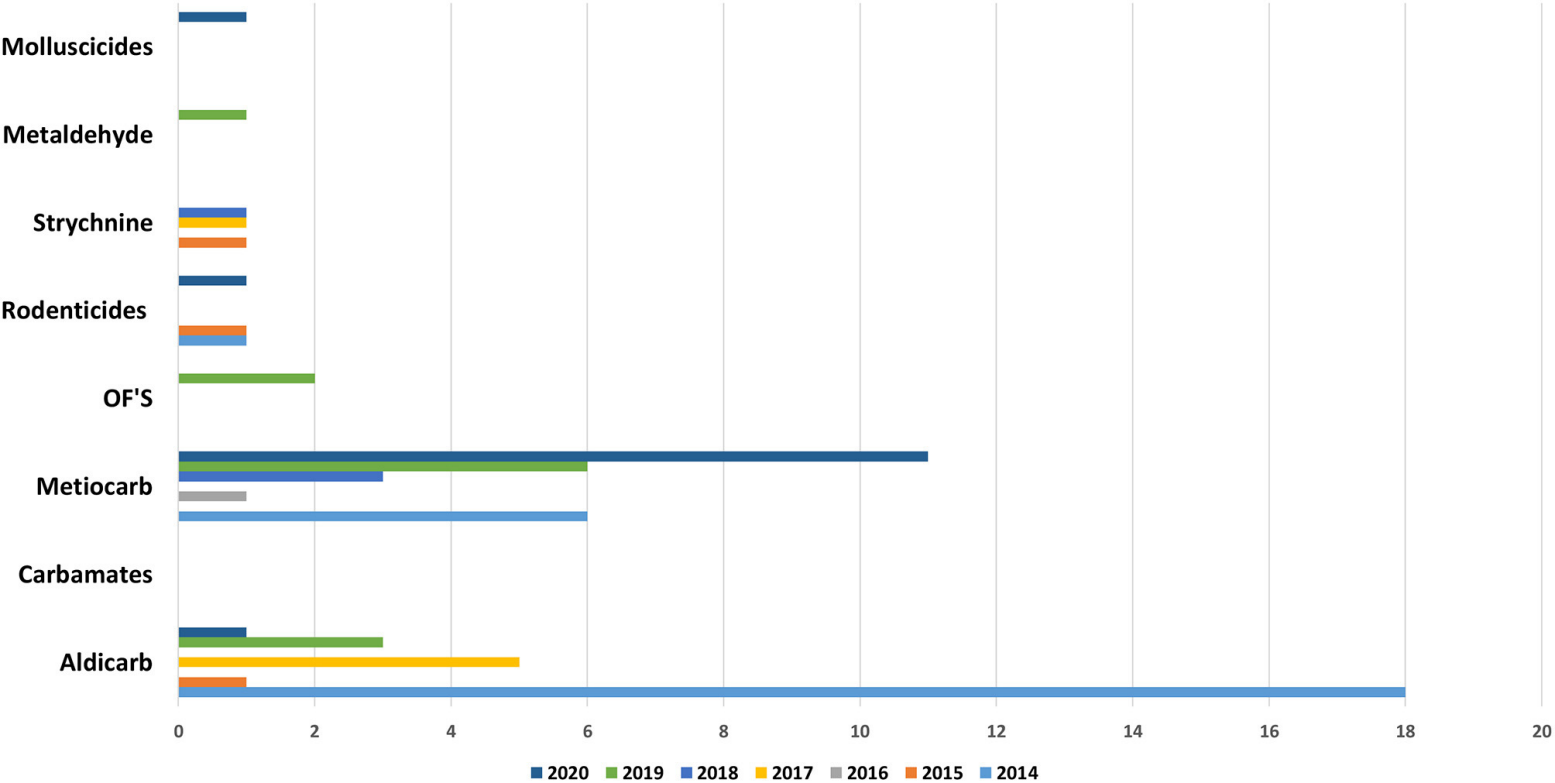
Distribution of substances in baits per year.

**Figure 10:**
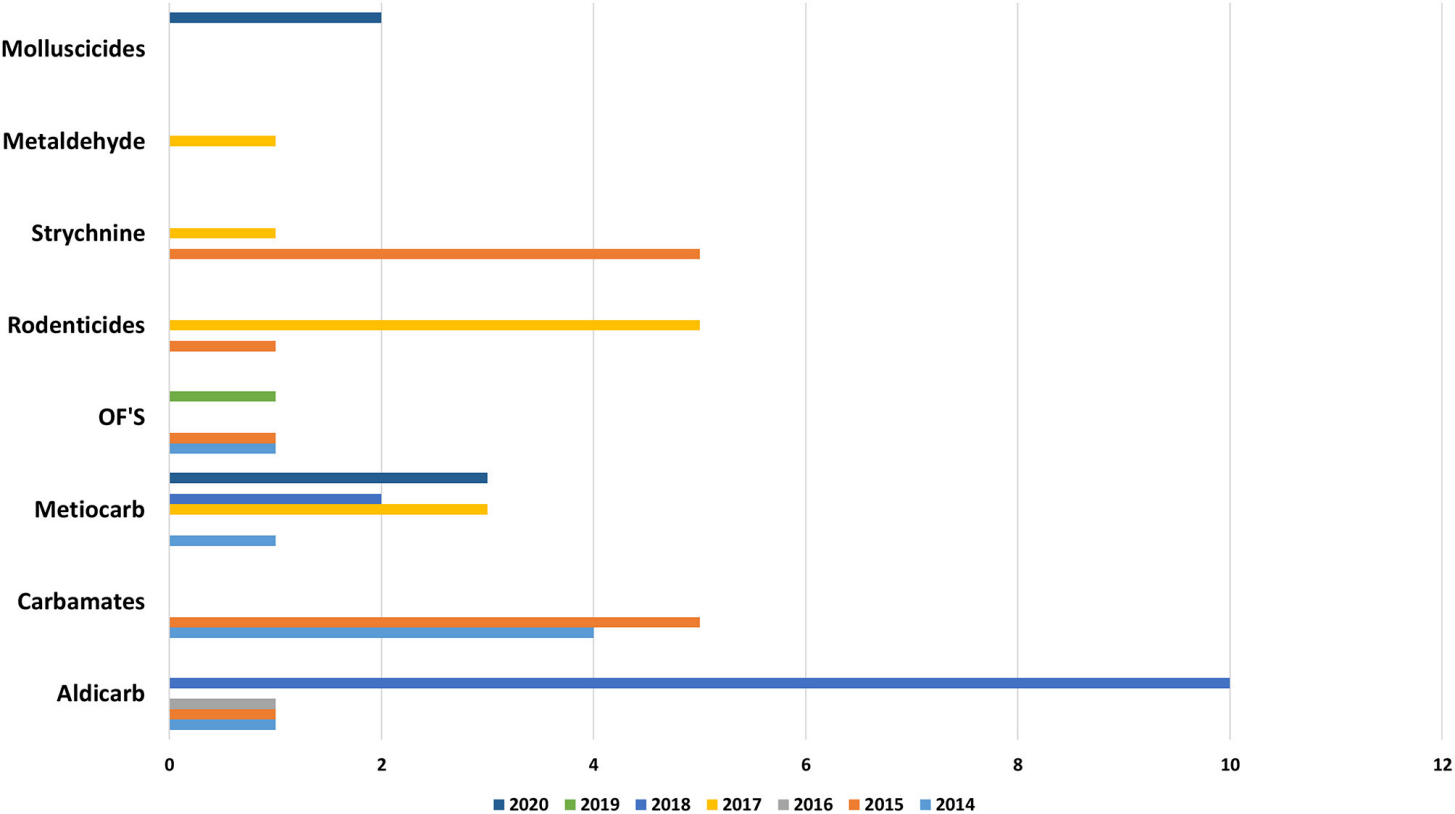
Substances in wildlife per year.

Anticoagulants pesticides are substances that are widely used for pest control. Poisoning due to these compounds in domestic animals (dogs, horses, and cats) and wild animals (foxes and bird of prey) must occur by occasional consumption of anticoagulants baits and contaminated prey (secondary poisoning). Liver is the tissue of choice for detection of these molecules (27). These substances interfere with vitamin K- mediated synthesis of blood clotting factors in the liver. Death usually occurs several days after ingestion of the rodenticides and the residues remaining are usually small. Our results (Figures 5, 8–10) shows that anticoagulants still cause poisoning in domestic and wild species.

We are aware about the analytical technique (TLC) used because it could not allow to make an exact quantification of the substances present and because LOD could not be as low as desirable. So, it could happen that in some cases the substance could be present at concentration <LOD and be reported as undetected (false negative). If this occurs our results are lower than the real scenario of pesticide poisoning in animals in the country.

In summary this study gives us a picture of the occurrence and frequency of pesticide poisoning in domestic and wild animals in Portugal since 2014 to 2020, however these results were only those obtained in LFT-FMV and probably not an exact image of the portuguese situation. In any case, as LFT-FMV is one of the most referenced laboratories nationwide by several projects taking place in country, most of the samples are sent to LFT-FMV so this study gives a good idea of the national panorama.

Ingestion stills the most common way that pesticides get into non-target organisms, and most often occurs through baits left in order to kill these animals. In Europe, this illegal practice represents one of the biggest conservation problems for some endangered species, often becoming the main cause of non-natural death. Species protection projects are an asset in this matter because without them it wouldn't be possible to monitor the areas of Portugal where the protected species are located and, consequently, analyses wouldn't be carried out, and we wouldn't have a real picture of the poisonings in the country.

So it is very important trying to understand what motivates these poisonings because only then will be possible to work on solutions for the problem and to take preventive and other prohibitive measures that make possible to identify who buys toxic substances and where, when and how this type of substances are acquired.

## Data Availability Statement

The raw data supporting the conclusions of this article will be made available by the authors, without undue reservation.

## Author Contributions

All authors had been evolved in the work and in the manuscript elaboration.

## Conflict of Interest

The authors declare that the research was conducted in the absence of any commercial or financial relationships that could be construed as a potential conflict of interest.
